# Supplementary material for: Reporting Quality of Studies Developing and Validating Melanoma Prediction Models: An Assessment Based on the TRIPOD Statement
Source: Healthcare (Basel). 2022 Jan 26;10(2):238. doi: 10.3390/healthcare10020238 (PMC8871554; doi:10.3390/healthcare10020238)
Supplement: Supplementary file 1 [file healthcare-10-00238-s001.zip › healthcare-1544773-supplementary.pdf]

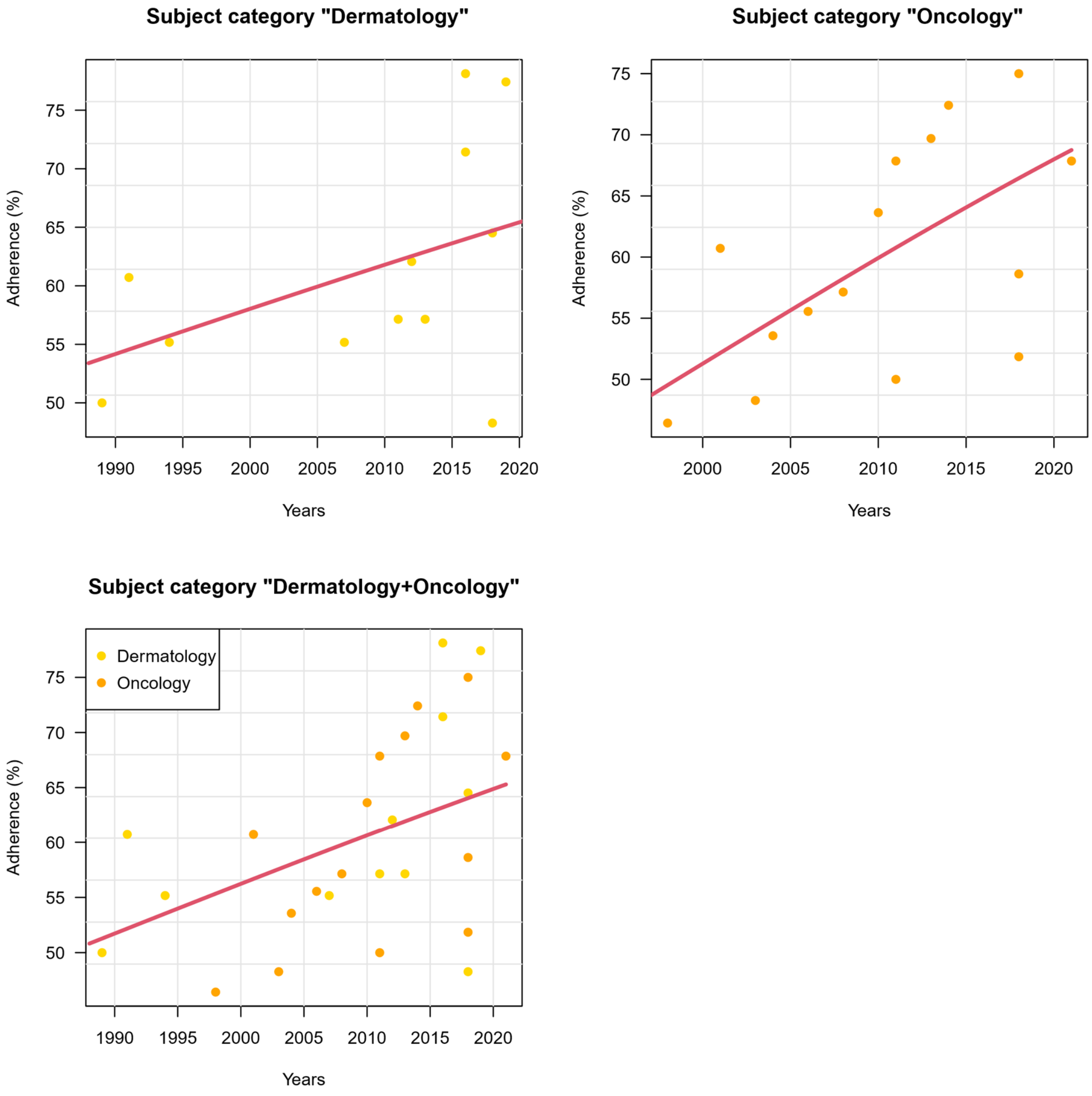

Figure S1: Relationship between TRIPOD adherence and publication year based on studies from different subject categories. Red lines represent the predicted mean curve from a beta regression model. 1) Only studies of the subject category "Dermatology" (N=12), $p<0.05$; 2) only studies of the subject category "Oncology" (N=15), $p<0.01$; 3) studies of both subject categories "Dermatology" and "Oncology" (N=27), $p<0.01$.

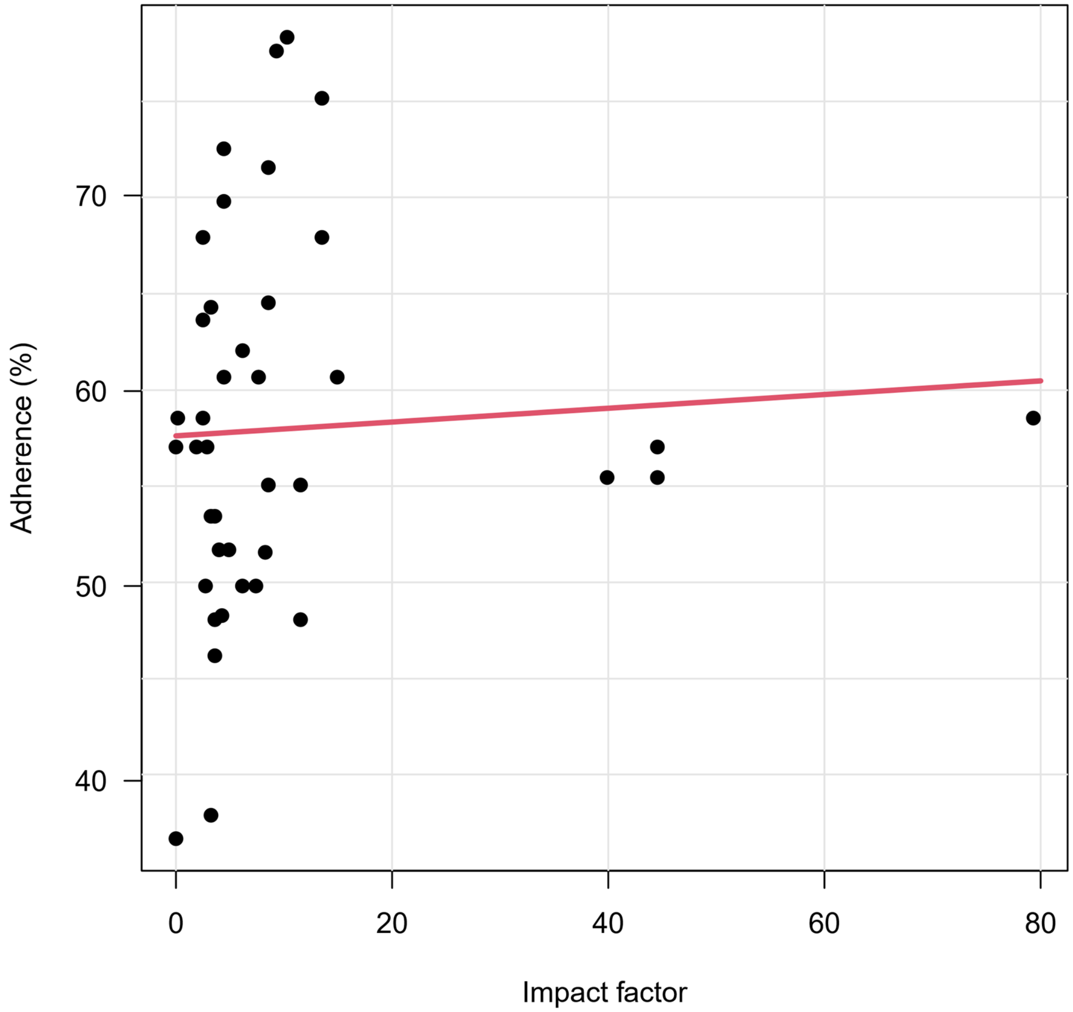

Figure S2: Relationship between TRIPOD adherence and impact factor. Red line represents the predicted mean curve from a beta regression model based on 40 studies (two studies were excluded, see text)
